# Effect of Qualifying Atherosclerotic Cardiovascular Disease Diagnosis Proximity on Cardiovascular Risk and Benefit of Empagliflozin in the EMPA-REG OUTCOME Trial

**DOI:** 10.1016/j.cjco.2024.01.013

**Published:** 2024-02-09

**Authors:** Ayodele Odutayo, Bernard Zinman, Christoph Wanner, Isabella Zwiener, Søren S. Lund, Stefan Hantel, David Fitchett, Jacob A. Udell, EMPA-REG OUTCOME Trial Investigators

**Affiliations:** a Department of Medicine, University of Toronto, Toronto, Ontario, Canada; b Cardiovascular Division, Women’s College Hospital and Peter Munk Cardiac Centre, University Health Network, Toronto, Ontario, Canada; c Division of Nephrology, University Health Network, Toronto, Ontario, Canada; d Lunenfeld-Tanenbaum Research Institute, Mount Sinai Hospital, Toronto, Ontario, Canada; e Department of Internal Medicine I, Nephrology, University Hospital Würzburg, Würzburg, Germany; f Clinical Trial Service Unit, Department of Population Health, University of Oxford, Nuffield, Oxford, UK; g Boehringer Ingelheim Pharma GmbH & Co. KG, Ingelheim, Germany; h Boehringer Ingelheim International GmbH, Ingelheim, Germany; i Boehringer Ingelheim Pharma GmbH & Co. KG, Biberach, Germany; j St. Michael’s Hospital, Division of Cardiology, Toronto, Ontario, Canada

## Abstract

**Background:**

In patients with type 2 diabetes mellitus (T2DM), a history of an ischemic event is associated with increased risk for cardiovascular (CV) disease. Whether patients with T2DM and a recent atherothrombotic diagnosis benefit from early intervention with a sodium-glucose co-transporter 2 inhibitor is unknown.

**Methods:**

This study is a secondary analysis of the **Empa**gliflozin Cardiovascular **Outcome** Event Trial in Type 2 Diabetes Mellitus Patients–**R**emoving **E**xcess **G**lucose (EMPA-REG OUTCOME), which compared empagliflozin to placebo in adults with T2DM and atherosclerotic CV disease (ASCVD). Participants were categorized based on the time since their last qualifying ASCVD diagnosis (≤ 1 year vs > 1 year). Qualifying ASCVD diagnoses included ischemic or hemorrhagic stroke, myocardial infarction, coronary artery disease, and peripheral artery disease. The primary outcome was a composite of CV death, nonfatal myocardial infarction, or nonfatal stroke.

**Results:**

A total of 6796 participants (n = 4547 empagliflozin, n = 2249 placebo) were included. Median time since the last qualifying ASCVD diagnosis was 3.8 years (quartile 1-quartile 3: 1.5-7.6), and most qualifying diagnoses occurred > 1 year before randomization (≤ 1 year, n = 1214; > 1 year, n = 5582). Empagliflozin reduced the incidence of the primary outcome irrespective of the time since the last qualifying ASCVD diagnosis (≤ 1 year: hazard ratio 0.82, 95% confidence interval: 0.57-1.16; vs > 1 year: hazard ratio 0.85, 95% confidence interval: 0.72-1.00; *P* for interaction = 0.84). Results were similar for the composite of CV death or hospitalization for heart failure.

**Conclusions:**

Empagliflozin improved CV outcomes in participants with T2DM, irrespective of the time since the last qualifying ASCVD diagnosis at randomization. Prospective trials are necessary to investigate the use of sodium-glucose co-transporter 2 inhibitors at the time of an acute ASCVD event.

**Trial Registration:**

EMPA-REG OUTCOME (Clinicaltrials.gov identifier: NCT01131676).

## Graphical abstract

**Figure:**
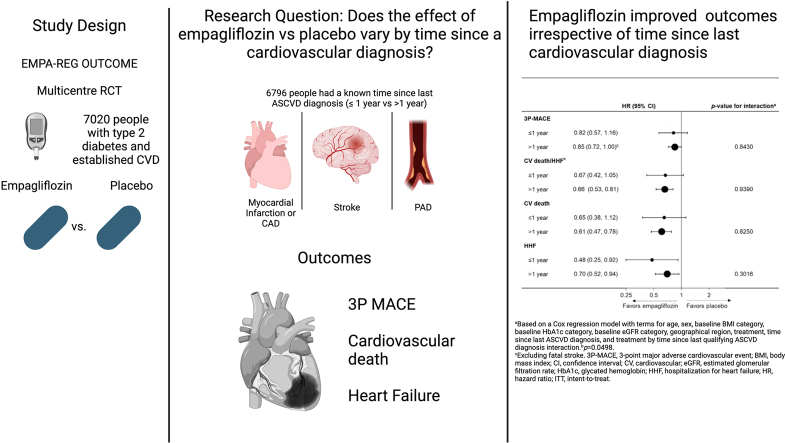

## Résumé

**Contexte:**

Chez les patients atteints de diabète de type 2 (DT2), des antécédents d’accidents ischémiques sont associés à un risque accru de maladie cardiovasculaire (CV). On ignore si une intervention précoce par un inhibiteur du cotransporteur sodium-glucose de type 2 pourrait être bénéfique pour les patients atteints de DT2 ayant récemment reçu un diagnostic d’athérothrombose.

**Méthodologie:**

Cette étude est une analyse secondaire de l’essai EMPA-REG OUTCOME (***Empa****gliflozin Cardiovascular****Outcome****Event Trial in Type 2 Diabetes Mellitus Patients–****R****emoving****E****xcess****G****lucose*), qui visait à comparer l’empagliflozine à un placebo chez des adultes atteints de DT2 et d’une maladie CV athéroscléreuse. Les participants ont été répartis selon le temps écoulé depuis leur plus récent diagnostic de maladie CV athéroscléreuse admissible (≤ 1 an *vs* > 1 an). Les diagnostics de maladies CV athéroscléreuses admissibles comprenaient un accident vasculaire cérébral ischémique ou hémorragique, un infarctus du myocarde, une coronaropathie et une artériopathie périphérique. Le critère d’évaluation principal était composé des décès d’origine CV, des infarctus du myocarde non mortels et des accident vasculaire cérébral non mortels.

**Résultats:**

Au total, 6796 participants (n = 4547 pour l’empagliflozine, n = 2249 pour le placebo) ont été inclus. Le temps écoulé médian depuis le diagnostic le plus récent de maladie CV athéroscléreuse admissible était de 3,8 ans (quartile 1-quartile 3 : 1,5-7,6). La plupart des diagnostics admissibles avaient été posés plus de 1 an avant la répartition aléatoire (≤ 1 an, n = 1214; > 1 an, n = 5582). L’empagliflozine a réduit la fréquence des événements constituant le critère d’évaluation principal, sans égard au temps écoulé depuis le plus récent diagnostic de maladie CV athéroscléreuse admissible (≤ 1 an : rapport des risques instantanés 0,82, intervalle de confiance à 95 % : 0,57-1,16; *vs* > 1 an : rapport des risques instantanés 0,85, intervalle de confiance à 95 % : 0,72-1,00; *p* pour l’interaction = 0,84). Les résultats étaient comparables à ceux observés pour le critère composé des décès d’origine CV et des hospitalisations pour insuffisance cardiaque.

**Conclusions:**

L’empagliflozine a amélioré les issues CV chez les patients atteints de DT2 sans égard au temps écoulé depuis le plus récent diagnostic de maladie CV admissible au moment de la répartition aléatoire. Des essais prospectifs sont nécessaires pour étudier l’utilisation des inhibiteurs du cotransporteur sodium-glucose de type 2 lorsqu’une manifestation de maladie CV athéroscléreuse aiguë survient.

**Inscription de l’essai:**

EMPA-REG OUTCOME (numéro d’identification dans clinicaltrials.gov : NCT01131676).

In patients with type 2 diabetes mellitus (T2DM), a history of an ischemic event is associated with an increased risk for the composite 3-point major adverse cardiovascular event (3P-MACE) endpoint of recurrent myocardial infarction (MI), stroke, or cardiovascular (CV) death. However, as patients achieve longer event-free survival from an acute MI or stroke, a progressive decrease occurs in the risk of recurrent atherothrombotic events and acute heart failure.^1^ A similar phenomenon has been observed for patients following a diagnosis of, and intervention for, coronary artery disease (CAD).^2^

Sodium-glucose co-transporter 2 (SGLT2) inhibitors are newly established treatments for reducing cardiovascular events in people with T2DM at high CV risk, chronic kidney disease, and heart failure.^3^, ^4^, ^5^ Specific to adults with T2DM, with or without established atherosclerotic CV disease (ASCVD), SGLT2 inhibitors reduced the incidence of CV death or hospitalization for heart failure (HHF) by 32% and reduced the incidence of risk of 3P-MACE by 10%.^6^ Whether identifying patients with T2DM and a recent atherothrombotic event as a target population for early intervention with an SGLT2 inhibitor will reduce recurrent CV events is unknown.

The **Empa**gliflozin Cardiovascular **Outcome** Event Trial in Type 2 Diabetes Mellitus Patients–**R**emoving **E**xcess **G**lucose (EMPA-REG OUTCOME) trial was a large CV outcome study in participants with T2DM and ASCVD that demonstrated empagliflozin improved outcomes for the primary composite of 3P-MACE (defined as time to cardiovascular death, nonfatal MI, or nonfatal stroke), time to HHF, time to cardiovascular death, and time to all-cause mortality.^7^^,^^8^ The aim of the present analysis was to explore the association of time since the last qualifying ASCVD diagnosis at randomization with the risk of cardiovascular outcomes and the consistency of benefit of empagliflozin across time from last qualifying ASCVD diagnosis.

## Materials and Methods

### Study design

The study design has been described previously.^8^^,^^9^ In brief, EMPA-REG OUTCOME was a randomized, double-blind, placebo-controlled, multinational trial. Participants entered a 2-week, open-label, placebo run-in prior to randomization to empagliflozin 10 mg, empagliflozin 25 mg, or placebo once-daily, in addition to standard of care, according to the discretion of the investigator for T2DM and CV risk management. Investigators were encouraged to treat CV risk factors to achieve optimal standard of care according to local guidelines. The trial continued until ≥ 691 participants experienced an adjudicated 3P-MACE. CV outcome events and deaths were prospectively adjudicated by a Clinical Events Committee and have been described previously.^8^ Participants who prematurely discontinued study medication were followed for ascertainment of CV outcomes and vital status. An independent ethics committee or institutional review board approved the clinical protocol at each participating centre. All participants provided their written informed consent prior to participation.

### Participants

Eligible participants were adults with T2DM (treatment-naïve patients: glycated hemoglobin [HbA1c] 7%-9%; participants on stable glucose-lowering therapy: HbA1c 7%-10%), a body-mass index of ≤ 45 kg/m^2^, prevalent cardiovascular disease typically of atherosclerotic origin (prior history of MI, CAD, peripheral artery disease [PAD] and/or prior stroke; Supplemental Table S1), and an estimated glomerular filtration rate (eGFR) according to the Modification of Diet in Renal Disease formula of ≥ 30 mL/min per 1.73 m^2^. Participants with acute coronary syndrome, stroke (either ischemic or hemorrhagic). or transient ischemic attack < 2 months prior to informed consent were excluded (Supplemental Table S1).

Participants were categorized into subgroups based on the time since their last qualifying ASCVD diagnosis (≤ 1 year vs > 1 year). Qualifying ASCVD diagnosis included any of the following: ischemic or hemorrhagic stroke, MI, documented CAD (both single and multivessel), or documented PAD. Documented CAD was defined as single-vessel CAD if ≥ 50% luminal narrowing was present during angiography by coronary or multi-slice computed tomography (CT) not subsequently successfully revascularized, with a positive noninvasive stress test for ischemia and/or hospital discharge for unstable angina ≤ 12 months prior to consent. Multivessel CAD was defined as the presence of significant stenosis (≥ 50% luminal narrowing during angiography by coronary or multi-slice CT), previous revascularization (percutaneous transluminal coronary angioplasty with or without stenting or coronary artery bypass graft > 2 months prior to consent), or the combination of revascularization in one major coronary artery and significant stenosis (≥ 50% luminal narrowing) in another major coronary artery. Documented PAD was defined as a history of limb angioplasty, stenting, or bypass surgery, limb or foot amputation due to circulatory insufficiency, evidence of significant peripheral artery stenosis (> 50% on angiography, or > 50% or hemodynamically significant via noninvasive methods) in 1 limb, or ankle brachial index < 0.9 in either or both ankles. Important to note is that participants with a qualifying ASCVD diagnosis of multivessel CAD based on coronary angiography or CT angiography, single-vessel CAD or PAD within 2 months of consent were eligible for inclusion (Supplemental Table S1).

### Outcomes and statistical analyses

The prespecified CV outcomes included the following: (i) 3P-MACE; (ii) a composite of CV death or HHF; (iii) CV death; and (iv) HHF. Analyses were performed in participants treated with ≥ 1 dose of study drug. The analyses presented here on the qualifying ASCVD diagnosis were post hoc. In all analyses, the 2 empagliflozin dose groups (10 mg and 25 mg) were pooled. Categorical variables are summarized as absolute numbers and percentages, and continuous variables are summarized as means and standard deviations, or medians and interquartile ranges, as appropriate. We constructed Kaplan-Meier event rates and/or curves for CV outcomes determined for participants at ≤ 1 year and > 1 year since last qualifying ASCVD diagnosis and treatment with empagliflozin or placebo.

A Cox proportional hazards model was used to examine the association between time since the last qualifying ASCVD diagnosis (≤ 1 year vs > 1 year) and the risk of CV outcomes in the placebo arm, with adjustment for age, sex, as well as stratification factors incorporated into the randomization sequence: baseline body mass index, baseline HbA1c, baseline eGFR, and geographic region. This adjustment variable included in the multivariable Cox model is consistent with the primary report of the EMPA-REG OUTCOME trial. These analyses were repeated in the pooled empagliflozin intervention groups. A Cox proportional hazards model was then used to compare the effect of empagliflozin treatment vs placebo on the risk of CV events in the subgroups of participants based on the time since the qualifying ASCVD diagnosis (≤ 1 year and > 1 year), with adjustment for age, sex, baseline body mass index, baseline HbA1c, baseline eGFR, geographic region, and treatment with empagliflozin or placebo. Heterogeneity in the effect of empagliflozin vs placebo based on the time since last qualifying ASCVD diagnosis was assessed by introducing a treatment-by-subgroup interaction term in the models. In a sensitivity analysis, the Cox regression model included time since last qualifying ASCVD diagnosis as a continuous variable, instead of the categorical one. A further test for interaction was conducted between treatment, time since the last qualifying ASCVD diagnosis (≤ 1 year and > 1 year), and type of qualifying ASCVD diagnosis (stroke, PAD, and MI and/or CAD).

## Results

### Baseline characteristics according to the time since the last qualifying ASCVD diagnosis

**Figure 1:**
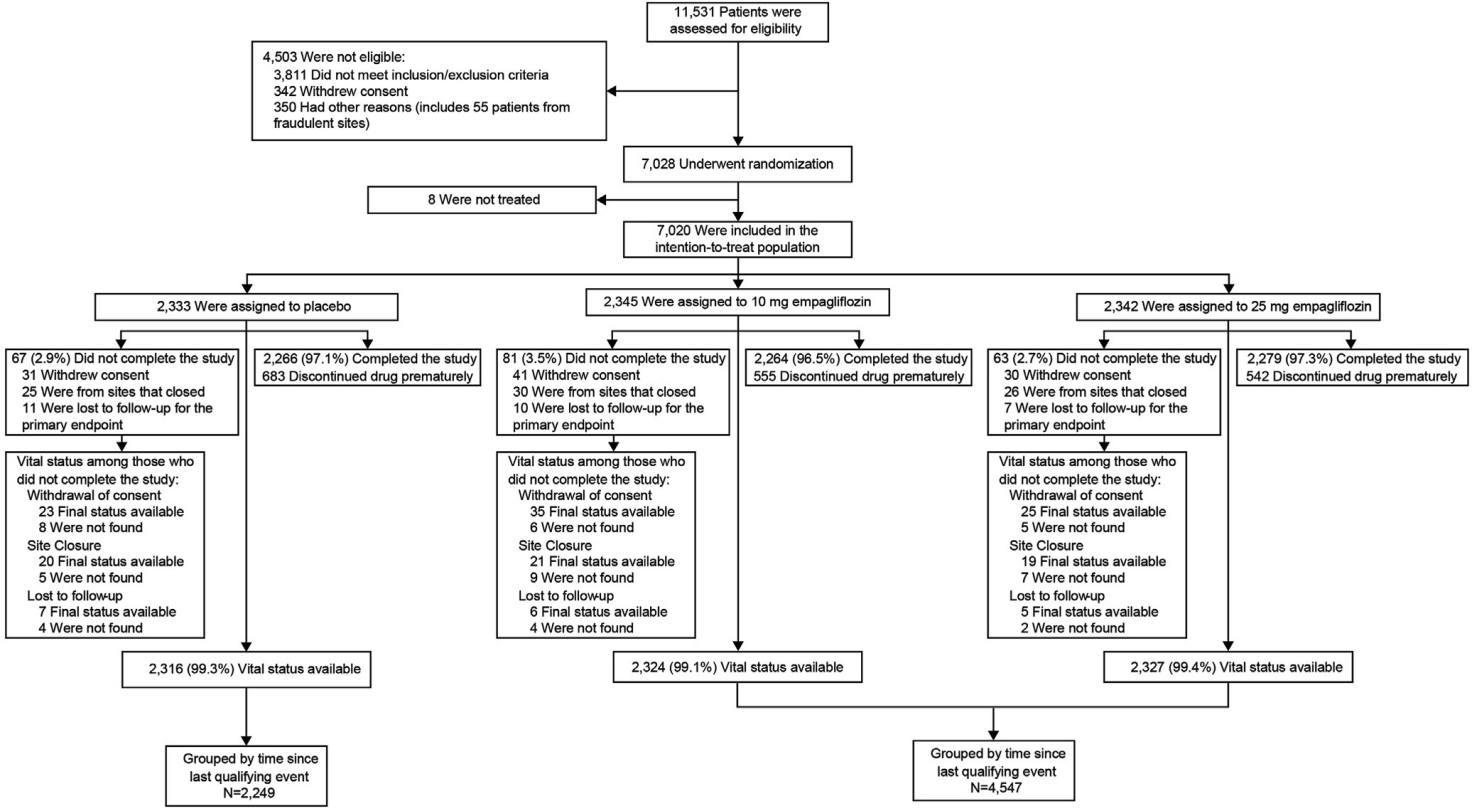
Consolidated standards of reporting trials (CONSORT) diagram.

Of 7028 participants randomized to study treatment from September 2010 to April 2013, 7020 participants at 590 sites in 42 countries received ≥ 1 dose of study drug (placebo, n = 2333; empagliflozin 10 mg, n = 2345; empagliflozin 25 mg, n = 2342) and were included in the primary analysis.^8^^,^^10^ The median duration of treatment was 2.6 years, and the median follow-up time was 3.1 years, with 97% of participants completing the trial. A total of 6796 participants (97%; n = 4547 empagliflozin, n = 2249 placebo) were able to be categorized by time since last qualifying ASCVD diagnosis and were included in this analysis (Fig. 1). Baseline demographics and characteristics of the study population, categorized by time since last qualifying ASCVD diagnosis, are shown in Table 1.

**Table 1:** Baseline demographics and characteristics according to time since last qualifying atherosclerotic cardiovascular disease (ASCVD) diagnosis

Parameter	Time since last qualifying ASCVD diagnosis	
	≤ 1 y (n = 1214)	> 1 y (n = 5582)
Male	806 (66.4)	4051 (72.6)
Age, y	61.7 ± 9.3	63.4 ± 8.4
Race		
White	834 (68.7)	4082 (73.1)
Black or African American	93 (7.7)	257 (4.6)
Asian	274 (22.6)	1192 (21.4)
Other	13 (1.1)	50 (0.9)
BMI, kg/m^2^	29.80 ± 5.25	30.80 ± 5.26
eGFR, mL/min per 1.73 m^2^	77.10 ± 23.19	73.44 ± 20.96
HbA1c, %	8.07 ± 0.88	8.07 ± 0.84
SBP, mm Hg	135.0 ± 17.4	135.5 ± 16.9
DBP, mm Hg	77.3 ± 10.1	76.6 ± 9.8
LDL-C, mmol/L	2.28 ± 0.95	2.20 ± 0.92
Current smoker	152 (12.5)	749 (13.4)
T2DM diagnosis > 10 y	644 (53.0)	3,234 (57.9)
Glucose-lowering therapy		
Metformin	874 (72.0)	4148 (74.3)
Sulfonylurea	514 (42.3)	2402 (43.0)
Insulin	554 (45.6)	2726 (48.8)
Antihypertensive medication	1114 (91.8)	5337 (95.6)
Lipid-lowering drugs	935 (77.0)	4561 (81.7)
Anticoagulants∗	1061 (87.4)	4993 (89.4)
Antiplatelets	1035 (85.3)	4799 (86.0)

Data are n (%) or mean ± standard deviation in participants treated with ≥ 1 dose of study drug. Placebo and empagliflozin groups have been pooled. BMI, body mass index; DBP, diastolic blood pressure; eGFR, estimated glomerular filtration rate; HbA1c, glycated hemoglobin; LDL-C, low-density lipoprotein cholesterol; SBP, systolic blood pressure; T2DM, type 2 diabetes mellitus.

∗ Excluding heparin.

Demographic and clinical characteristics and concomitant medication use were similar between the placebo and empagliflozin groups across the qualifying ASCVD diagnosis subgroups. Participants in the > 1 year subgroup typically had a more adverse cardiovascular risk profile, and they were more likely to be male, were older, had a longer duration of T2DM, had a lower eGFR, and were more likely to use CV protective therapies such as antihypertensive or lipid-lowering drugs than were participants in the ≤ 1 year subgroup. The distribution of time since last qualifying ASCVD diagnosis is shown in Supplemental Figure S1. The median time since the last qualifying ASCVD diagnosis was 3.8 years (quartile 1-quartile 3: 1.5-7.6), and most participants had a last qualifying ASCVD diagnosis at > 1 year prior to randomization (≤ 1 year subgroup: n = 1214 [17.9%]; > 1 year subgroup: n = 5582 [82.1%]). A total of 610 participants (9.0%; 193 placebo participants, and 417 empagliflozin participants) had a qualifying event 2-6 months prior to randomization. The most common types of qualifying ASCVD diagnosis were PAD (33.7%), multivessel CAD (26.9%), and stroke (21.0%) in the ≤ 1 year subgroup, and multivessel CAD (41.5%), MI (18.8%), and stroke (17.6%) in the > 1 year subgroup (Table 2).

**Table 2:** Type of last qualifying atherosclerotic cardiovascular disease (ASCVD) diagnosis, overall and by treatment group (modified intent-to-treat population)

Last qualifying ASCVD diagnosis	≤ 1 y since last qualifying ASCVD diagnosis (n = 1214)			> 1 y since last qualifying ASCVD diagnosis (n = 5582)			Overall (N = 6796)
	Placebo (n = 373)	Empagliflozin (n = 841)	Total (N = 1214)	Placebo (n = 1876)	Empagliflozin (n = 3706)	Total (N = 5582)	
History of ischemic or hemorrhagic stroke	86 (23.1)	169 (20.1)	255 (21.0)	331 (17.6)	650 (17.5)	981 (17.6)	1236 (18.2)
History of MI	27 (7.2)	97 (11.5)	124 (10.2)	339 (18.1)	708 (19.1)	1047 (18.8)	1171 (17.2)
Multivessel CAD	95 (25.5)	232 (27.6)	327 (26.9)	792 (42.2)	1522 (41.1)	2314 (41.5)	2641 (38.9)
Single-vessel CAD	32 (8.6)	67 (8.0)	99 (8.2)	154 (8.2)	330 (8.9)	484 (8.7)	583 (8.6)
PAD	133 (35.7)	276 (32.8)	409 (33.7)	260 (13.9)	496 (13.4)	756 (13.5)	1165 (17.1)

Data are n (%) and represent participants treated with ≥ 1 dose of study drug. CAD, coronary artery disease; MI, myocardial infarction; PAD, peripheral artery disease.

### CV risk according to the time since the last qualifying ASCVD diagnosis

Among all participants in the placebo group, the time to 3P-MACE, CV death or HHF, CV death, and HHF alone was comparable in participants with a last qualifying ASCVD diagnosis ≤ 1 year before randomization vs > 1 year before randomization, although numerical differences were present (Supplemental Figs. S2 and S3). After multivariable adjustment, participants in the placebo arm who were enrolled with a qualifying ASCVD diagnosis ≤ 1 year from the time of randomization were not at a significantly higher risk for an outcome event during the trial, compared with participants enrolled with an older ASCVD diagnosis > 1 year before randomization (Table 3).

**Table 3:** Effect of timing of last qualifying atherosclerotic cardiovascular disease (ASCVD) diagnosis (≤ 1 y vs > 1 y) on cardiovascular (CV) events in placebo arm

Outcome	n with event / n analyzed (%)	HR (95% CI)∗	*P*†
**3P-MACE**			
Time since qualifying ASCVD diagnosis			
≤ 1 y	48/373 (12.9)	1.20 (0.88, 1.65)	0.2539
> 1 y (reference)	227/1876 (12.1)		
**HHF/CV death**†			
Time since qualifying ASCVD diagnosis			
≤ 1 y	31/373 (8.3)	1.14 (0.77, 1.68)	0.5206
> 1 y (reference)	158/1876 (8.4)		
**CV death**			
Time since qualifying ASCVD diagnosis			
≤ 1 y	22/373 (5.9)	1.11 (0.70, 1.76)	0.6620
> 1 y (reference)	110/1876 (5.9)		
**HHF**			
Time since qualifying ASCVD diagnosis			
≤ 1 y	18/373 (4.8)	1.43 (0.85, 2.41)	0.1789
> 1 y (reference)	73/1876 (3.9)		

CI, confidence interval; HHF, hospitalization for heart failure; HR, hazard ratio; 3P-MACE, 3-point major adverse cardiovascular events.

∗ Based on a Cox regression model with terms for age, time since last qualifying ASCVD diagnosis, sex, baseline body mass index category, baseline glycated hemoglobin (HbA1c) category, baseline estimated glomerular filtration rate category and geographic region.

† Excluding fatal stroke.

### Effect of empagliflozin according to the time since the last qualifying ASCVD diagnosis

**Figure 2:**
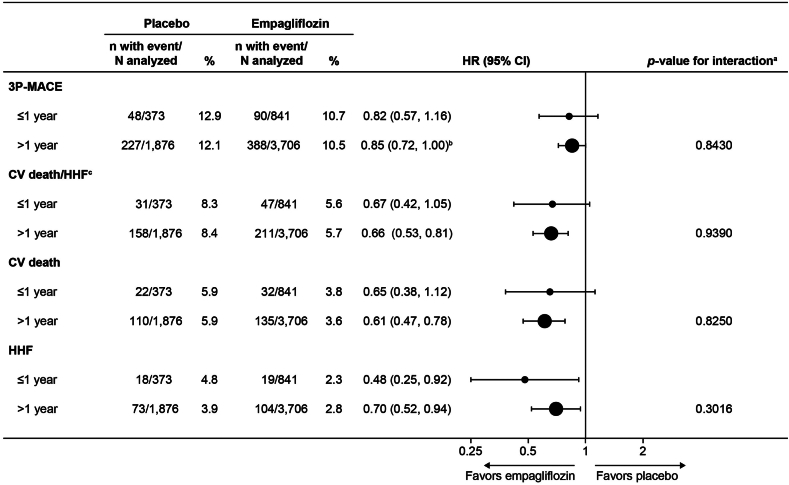
Cardiovascular (CV) outcomes, according to time since last qualifying atherosclerotic CV disease (ASCVD) diagnosis prior to randomization and treatment assignment. CI, confidence interval; HHF, hospitalization for heart failure; HR, hazard ratio; 3P-MACE, 3-point major adverse cardiovascular event. ^a^Based on a Cox regression model with terms for age, sex, baseline body mass index category, baseline glycated hemoglobin (HbA1c) category, baseline estimated glomerular filtration rate category, geographic region, treatment, time since last ASCVD diagnosis, and treatment by time since last qualifying ASCVD diagnosis interaction. ^b^*P* = 0.0498. ^c^Excluding fatal stroke.

For all of the assessed prespecified CV outcomes, treatment with empagliflozin was associated with a consistent and lower relative risk vs placebo, irrespective of time since last qualifying ASCVD diagnosis prior to randomization (Fig. 2 and Supplemental Fig. S4; all *P* for interactions ≥ 0.30). Results in 3P-MACE and the composite of CV death and HHF were similar regardless of the type of last qualifying ASCVD diagnosis (Supplemental Fig. S3; all *P* for interactions ≥ 0.05). The sensitivity analyses evaluating time since last qualifying ASCVD event as a continuous variable showed that no interaction occurred between treatment and time since last qualifying ASCVD diagnosis (all *P* for interactions > 0.05; otherwise, data not shown).

## Discussion

In the EMPA-REG OUTCOME trial of participants with T2DM and established ASCVD, approximately 1 in 6 participants were enrolled within a year from their last qualifying ASCVD diagnosis, and less than 10% were enrolled within 2-6 months of an ASCVD diagnosis. Most participants who enrolled within 1 year qualified with a diagnosis of PAD, multivessel CAD, or stroke. Relatively few participants (∼10%) were enrolled in the trial with a history of an acute MI within the prior year. In the placebo group, no significant association was present between the proximity of the last qualifying ASCVD diagnosis and the risk of 3P-MACE, CV death or HHF, or the individual endpoints of CV death and HHF. Moreover, empagliflozin demonstrated consistent risk reduction for these CV outcomes, irrespective of the time since the last qualifying ASCVD diagnosis. Results were consistent across all subgroups of qualifying ASCVD diagnosis for the assessed outcomes.

Our analysis demonstrates that although participants with T2DM enrolled in the EMPA-REG OUTCOME trial qualified based on a variety of ASCVD diagnoses, overall, fewer participants were enrolled early after a qualifying ASCVD diagnosis (median time since the last qualifying ASCVD diagnosis was close to 4 years). Participants with a relatively recent ASCVD diagnosis were not at higher risk for CV outcomes, compared with participants with a more distant ASCVD diagnosis. Multiple explanations for this observation are possible, including that participants with a last qualifying ASCVD diagnosis within 1 year had an apparent lower CV risk at baseline, and that participants in the trial could have had more than one diagnosis of ASCVD and a history of ASCVD diagnosis prior to the last qualifying ASCVD diagnosis, which may have negated any relative risk increase of a more recent diagnosis. Given this context, the finding of consistency in the benefit of empagliflozin across the spectrum of ASCVD diagnosis timing and last qualifying type of ASCVD diagnosis may not be surprising. Thus, our findings both support existing guideline recommendations on the use of SGLT2 inhibitors in patients with T2DM across a spectrum of ASCVD, and suggest an opportunity for further evaluation of the efficacy and safety of SGLT2 inhibitors among patients with acute ASCVD events.

Our findings are in contrast to those from a secondary prespecified analysis of the **D**apagliflozin **E**ffect on **C**ardiovascu**lar E**vents–**T**hrombolysis **i**n **M**yocardial **I**nfarction 58 (DECLARE-TIMI 58) trial, which compared the effect of dapagliflozin vs placebo in participants with a qualifying MI.^11^ In that study, 3584 of 17,160 participants had a qualifying MI prior to randomization, representing 21% of the study population. Within this subgroup, the magnitude of the relative and absolute risk reduction in 3P-MACE was larger among participants with a qualifying MI ≤ 12 months prior to randomization (adjusted hazard ratio [HR]: 0.66, 95% confidence interval [CI]: 0.42-1.03) or a qualifying MI that was within 12-24 months prior to randomization (HR: 0.42, 95% CI: 0.25-0.71) than in participants with a more distant qualifying MI (*P* for interaction = 0.0007). An interesting finding was that the evidence of benefit was less among participants with a qualifying MI 24-36 months prior to randomization (HR: 0.83, 95% CI: 0.50-1.40) or a qualifying MI that occurred more than 36 months prior to randomization (HR: 1.01, 95% CI: 0.82-1.23). No evidence of benefit was found of dapagliflozin vs placebo in participants with established ASCVD but no previous MI (HR: 0.98, 95% CI: 0.81-1.19).

The discordance between our results and those of the DECLARE-TIMI 58 analysis may relate to clinical and methodological differences. First, the subgroup of participants with a previous MI in the DECLARE-TIMI 58 trial were at higher CV risk compared with participants in the EMPA-REG OUTCOME trial with a more recent ASCVD diagnosis. Indeed, 17% of participants with a previous MI in the placebo group of the DECLARE-TIMI 58 analysis developed 3P-MACE,^11^ as compared to 12% of participants with a previous MI in the EMPA-REG OUTCOME trial.^12^ Furthermore, the fact that the data show no significant association between the proximity of the qualifying ASCVD diagnosis to study randomization in the placebo group, along with the primary outcome, suggests that EMPA-REG OUTCOME trial participants likely had relatively stable ASCVD at the time of randomization. Indeed, the definition of established CV disease in the EMPA-REG OUTCOME trial included imaging-based diagnoses of coronary and peripheral vascular disease without a corresponding hard CV event. Second, whereas the DECLARE-TIMI 58 analysis examined previous MI as a single type of qualifying event, we examined multiple types of qualifying ASCVD diagnoses in our main analysis, which may have contributed to clinical heterogeneity, thereby limiting ascertainment of effect modification. Third, the results of the secondary analysis of the DECLARE-TIMI 58 trial may be due to chance, and no association was present between the proximity of a qualifying MI and the relative risk reduction conferred by SGLT2 inhibitors. The use of SGLT2 inhibitors in the early period after an MI is being prospectively studied in 2 randomized controlled trials (RCTs; NCT04717986 and NCT04509674), and these studies will provide important insight on the efficacy of these medications during the acute phase of a CV event.

Beyond MI, SGLT2 inhibitors have demonstrated consistent relative risk reduction in CV events vs placebo in participants with heart failure, irrespective of the timing of the last HHF before randomization. In a meta-analysis of all available pivotal RCTs of SGLT2 inhibitors in 21,947 participants with heart failure, SGLT2 inhibitors showed a consistent relative risk reduction on the composite of CV death or first HHF in participants with an HHF within 12 months of randomization (HR 0.72, 95% CI: 0.65-0.80) vs in participants without an HHF within 12 months (HR: 0.80, 95% CI: 0.73-0.87; *P* for interaction = 0.52).^13^ The findings of this meta-analysis are also particularly important given that a secondary analysis of the **D**apagliflozin **a**nd **P**revention of **A**dverse Outcomes in **H**eart **F**ailure (DAPA-HF) trial published prior to the meta-analysis reported greater risk reduction in participants with an HHF within 12 months of randomization (> 1 year subgroup HR: 0.73, 95% CI: 0.54-0.99; ≤ 1 year subgroup HR: 0.64, 95% CI: 0.51-0.81).^14^ However, when DAPA-HF was included in the meta-analysis, along with other pivotal RCTs, these findings were not substantiated in the meta-analysis.

Our study has important limitations. First, this was a post hoc secondary analysis of the EMPA-REG OUTCOME trial, and our findings should be considered hypothesis-generating. Second, although 97% of participants in EMPA-REG OUTCOME trial had data on the timing of their qualifying ASCVD diagnosis to be included in our analysis, we had small sample sizes within specific strata of qualifying ASCVD diagnoses, and consequently, we had to categorize participants into subgroups based on timing across all qualifying ASCVD diagnoses, and not MI alone. Third, participants with an acute ASCVD event (acute coronary syndrome, stroke, transient ischemic attack) within 2 months of potential enrollment were excluded from the EMPA-REG OUTCOME trial. Fourth, details on stroke subtype were not collected in the EMPA-REG OUTCOME trial, and we could not distinguish between participants with an ischemic vs hemorrhagic stroke as their last qualifying event. Finally, few participants were studied within a year of a qualifying ASCVD diagnosis.

Taken together, empagliflozin, given in addition to standard of care, improved CV outcomes in participants with T2DM and ASCVD, irrespective of time since qualifying ASCVD diagnosis at randomization. This finding provides assurance to clinicians that the benefits of empagliflozin in terms of CV outcomes risk reduction apply to a broad population of patients with T2DM and atherosclerotic disease across the spectrum of time periods since an ASCVD diagnosis. Patients with acute MI may be a particularly important population in which to investigate the efficacy and safety of early initiation of an SGLT2 inhibitor.
