# Supplementary material for: Effect of Qualifying Atherosclerotic Cardiovascular Disease Diagnosis Proximity on Cardiovascular Risk and Benefit of Empagliflozin in the EMPA-REG OUTCOME Trial
Source: CJC Open. 2024 Feb 9;6(7):868–75. doi: 10.1016/j.cjco.2024.01.013 (PMC11252526; doi:10.1016/j.cjco.2024.01.013)
Supplement: Supplemental Figures and Tables [file mmc1.pdf]

SUPPLEMENTARY MATERIAL

Supplemental Table S1. Qualifying ASCVD Diagnosis and Minimum Elapsed Time for Eligibility

**Qualifying ASCVD Diagnosis**	**Minimum Elapsed Time for Eligibility***
1. Confirmed history of myocardial infarction	>2 months
2. Evidence of multivessel coronary artery disease, in 2 or more major coronary arteries, irrespective of the revascularization status:	
2.1 Either the presence of a significant stenosis (imaging evidence of at least 50% narrowing of the luminal diameter measured during a coronary angiography or a multi-sliced computed tomography angiography), in 2 or more major coronary arteries,	None
2.2 Or a previous revascularisation (percutaneous transluminal coronary angioplasty with or without stent, or coronary artery bypass grafting) at least 2 months ago, in 2 or more major coronary arteries,	>2 months
2.3 Or a previous revascularisation (percutaneous transluminal coronary angioplasty with or without stent, or coronary artery bypass grafting) at least 2 months ago, in 2 or more major coronary arteries,	> 2 months
3. Single vessel coronary artery disease	None
4. Unstable angina with confirmed evidence of coronary multivessel or single vessel disease	> 2 months
5. History of ischemic or haemorrhagic stroke	> 2 months
6. Presence of peripheral artery disease (symptomatic or not)	None

*Participants were excluded if they had an acute coronary syndrome, stroke or TIA within 2 months prior to informed consent

Supplemental Figure S1. Time of last qualifying ASCVD diagnosis prior to randomization.

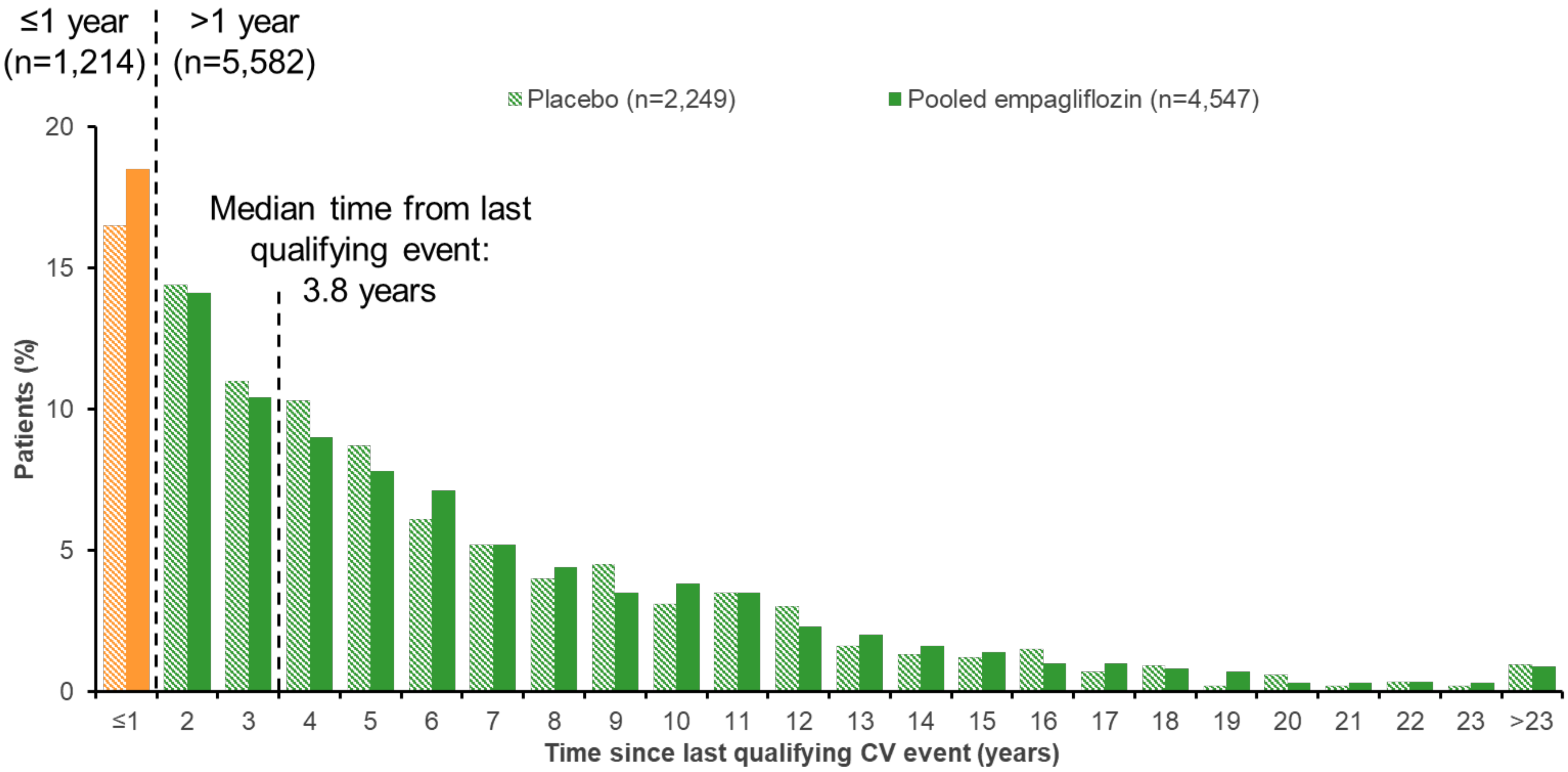

CV, cardiovascular

Supplemental Figure S2. Time to 3P-MACE, HHF and HHF/CV death, according to time since last qualifying ASCVD diagnosis prior to randomization, in placebo arm.

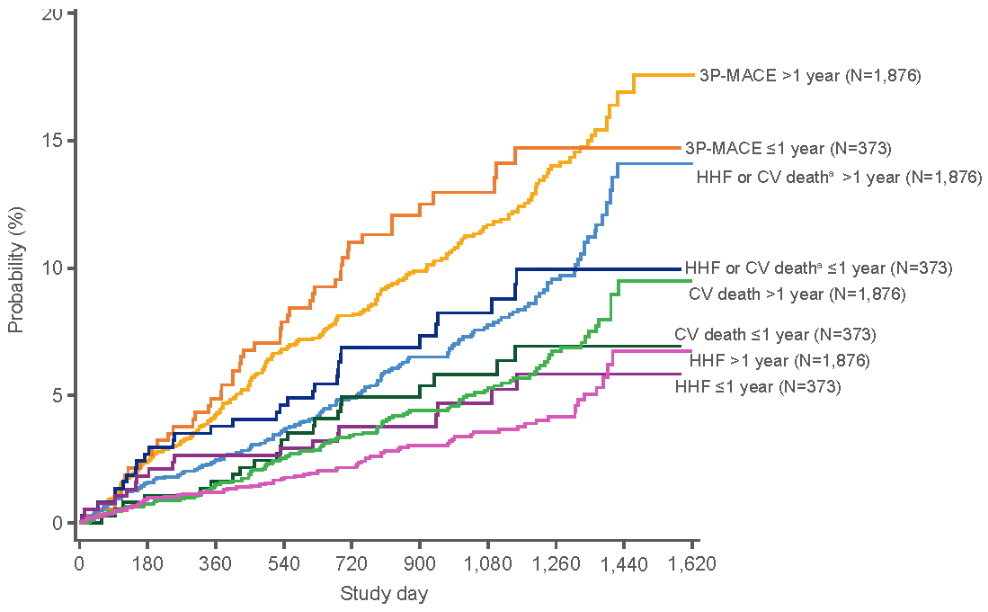

Participants at risk	0	180	360	540	720	900	1,080	1,260	1,440	1,620
3P-MACE										
≤1 year	373	359	348	335	295	194	162	90	14	
>1 year	1,876	1,815	1,766	1,701	1,506	1,122	945	612	143	1
HHF or CV death[a]										
≤1 year	373	358	350	344	306	208	172	97	16	
>1 year	1,876	1,831	1,797	1,754	1,554	1,155	979	641	143	1
CV death										
≤1 year	373	367	365	359	322	220	186	105	16	
>1 year	1,876	1,853	1,834	1,805	1,613	1,215	1,037	676	152	1
HHF										
≤1 year	373	358	350	344	306	208	172	97	16	
>1 year	1,876	1,831	1,797	1,754	1,554	1,155	979	641	143	1

[a]Excluding fatal stroke.

CV, cardiovascular; HHF, hospitalization for heart failure; 3P-MACE, 3-point major adverse cardiovascular events.

Supplemental Figure S3. Time to 3P-MACE (A) and composite of cardiovascular death (excluding fatal stroke) or HHF (B) by time since last qualifying ASCVD diagnosis and type of qualifying ASCVD diagnosis

A

Time and type of last qualifying event	Placebo			Empagliflozin				
	n with event/N analyzed	%	Incidence rate/1000 PY	n with event/N analyzed	%	Incidence rate/1000 PY	**HR (95% CI)[a]**	***P*-value for interaction**
**Last qualifying event: Stroke**								
≤1 year	17/86	19.8	81.6	27/169	16.0	61.9	0.80 (0.44, 1.47)	
>1 year	32/331	9.7	34.9	65/650	10.0	35.8	1.05 (0.69, 1.61)	
**Last qualifying event: PAD**								
≤1 year	13/133	9.8	37.6	21/276	7.6	28.7	0.73 (0.36, 1.45)	0.82
>1 year	37/260	14.2	53.7	69/496	13.9	51.9	0.95 (0.64, 1.42)	
**Last qualifying event: MI/single or multivessel CAD[b]**								
≤1 year	18/154	11.7	42.1	42/396	10.6	38.2	0.93 (0.54, 1.62)	
>1 year	158/1285	12.3	44.0	254/2560	9.9	34.8	0.79 (0.64, 0.96)	

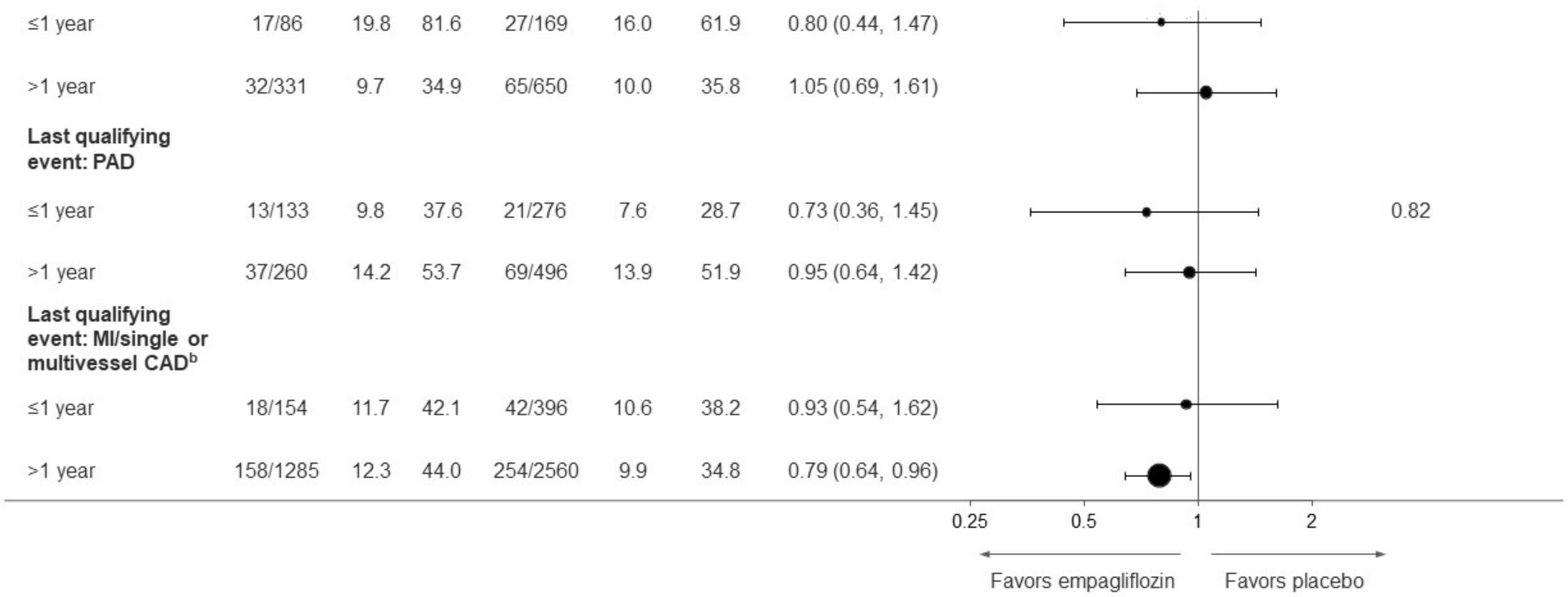

B

Time and type of last qualifying event	Placebo n with event/N analyzed	Placebo %	Placebo Incidence rate/1000 PY	Empagliflozin n with event/N analyzed	Empagliflozin %	Empagliflozin Incidence rate/1000 PY	HR (95% CI)[a]	*P*-value for interaction
**Last qualifying event: Stroke**								
≤1 year	10/86	11.6	45.9	11/169	6.5	23.9	0.57 (0.24, 1.34)	
>1 year	26/331	7.9	27.8	34/650	5.2	18.3	0.66 (0.40, 1.11)	
**Last qualifying event: PAD**								
≤1 year	11/133	8.3	31.3	12/276	4.3	16.10	0.48 (0.21, 1.08)	0.76
>1 year	28/260	10.8	39.6	44/496	8.9	32.0	0.77 (0.48, 1.24)	
**Last qualifying event: MI/single or multivessel CAD[b]**								
≤1 year	10/154	6.5	22.8	24/396	6.1	21.4	1.00 (0.48, 2.10)	
>1 year	104/1285	8.1	28.3	133/2560	5.2	17.8	0.62 (0.48, 0.81)	

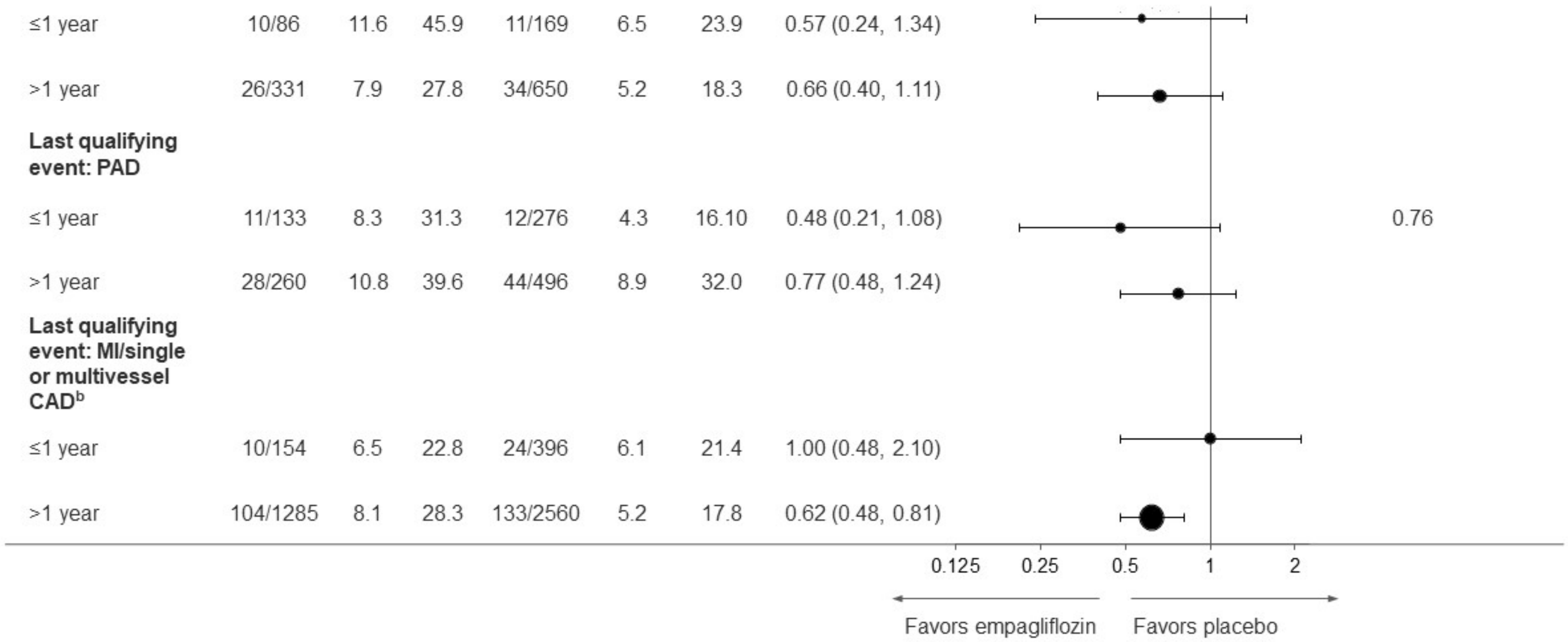

[a]Cox regression model with terms for age, sex, baseline BMI category, baseline HbA1c category, baseline eGFR category, geographical region, treatment, time and type of last qualifying pre−baseline ASCVD diagnosis and treatment by time and type of last qualifying pre−baseline ASCVD diagnosis interaction.

[b] These qualifying ASCVD diagnosis were combined due to low patient numbers.

3P-MACE, 3-point major adverse cardiovascular event; BMI, body mass index; CAD, coronary artery disease; CI, confidence interval; CV, cardiovascular; eGFR, estimated glomerular filtration rate; HbA1c, glycated hemoglobin; HR, hazard ratio; MI, myocardial infarction; PAD, peripheral artery disease; PY, patient year.

Supplemental Figure S4. Time to 3P-MACE according to time since last qualifying ASCVD diagnosis prior to randomization.

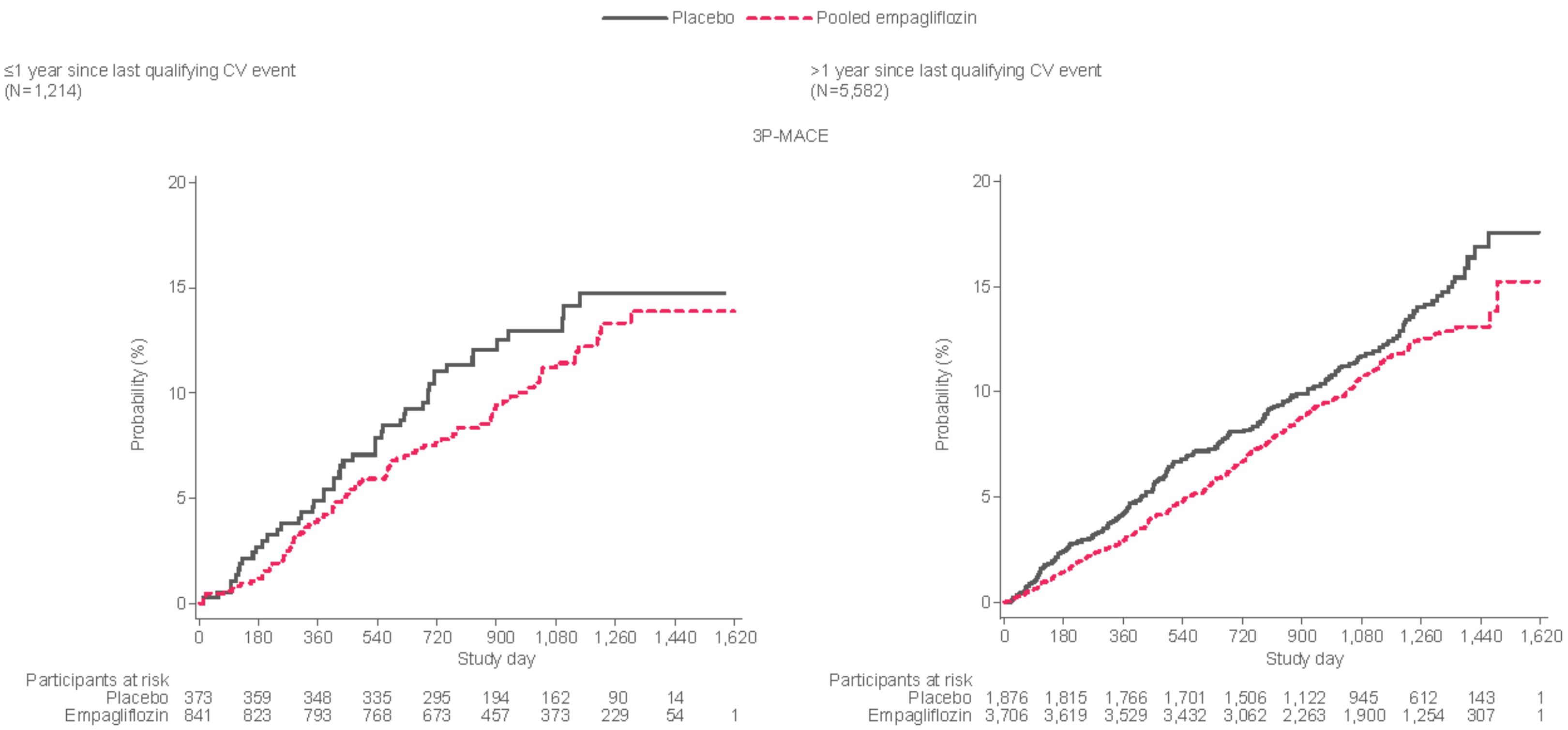

MACE, major adverse cardiovascular event; CV, cardiovascular; HHF, hospitalization for heart failure.
